# Predictors of do-not-attempt-resuscitation decisions in patients with infratentorial or large supratentorial intracerebral hemorrhages and consequences thereafter: a register-based, longitudinal study in Sweden

**DOI:** 10.1186/s12883-023-03056-2

**Published:** 2023-01-16

**Authors:** Amanda Soomägi, Adam Viktorisson, Katharina S. Sunnerhagen

**Affiliations:** Institute of Neuroscience and Physiology, Rehabilitation Medicine, Sahlgrenska Academy, University of Gothenburg, and the Sahlgrenska University Hospital, PO Box 430, Per Dubbsgatan 14, 3rd floor, SE 405 30 Gothenburg, Sweden

**Keywords:** Decision-making, Hemorrhagic stroke, Mortality, Prognosis, Resuscitation orders, DNAR, ICH

## Abstract

**Objectives:**

Do-not-attempt-resuscitation (DNAR) decisions for patients with infratentorial or large supratentorial intracerebral hemorrhages (ICHs) pose clinical and ethical challenges. We aimed to investigate factors associated with DNAR decisions in patients with infratentorial or large (≥30 mL) supratentorial ICH, and differences in complications, treatment, and mortality.

**Materials & methods:**

This longitudinal, observational study comprised all patients treated for ICH at three stroke units in Gothenburg, Sweden, between November 2014 and June 2019. Patients were identified in the local stroke register, and additional data were collected from medical records and national registries. Mortality rates were followed 1 year after incident ICHs. Factors associated with DNAR decisions, and one-year mortality were explored.

**Results:**

Of 307 included patients, 164 received a DNAR decision. Most (75%) decisions were made within 24 h. DNAR decisions were associated with higher age, pre-stroke dependency, stroke severity, and intraventricular hemorrhage. Patients without DNAR decisions received thrombosis prophylaxis, oral antibiotics, and rehabilitative evaluations more frequently. The one-year survival probability was 0.16 (95% confidence interval [CI] 0.11–0.23) in patients with DNAR decisions, and 0.87 (95% CI 0.81–0.92) in patients without DNAR decision. DNAR decisions, higher age, stroke severity, hematoma volume, and comorbidities were associated with increased one-year mortality. Thrombosis prophylaxis and living alone were associated with a lower hazard.

**Conclusion:**

The majority of DNAR decisions for patients with infratentorial or large supratentorial ICH were made within 48 h. Higher age, pre-stroke dependency, high stroke severity, and intraventricular hemorrhage predicted receiving a DNAR decision. DNAR decisions were strongly associated with increased short- and long-term mortality.

## Introduction

Intracerebral hemorrhage (ICH) accounts for 10–15% of stroke cases [1]. It is a severe disease, with one-year-mortality over 50% [2]. The strongest predictors for ICH mortality are hematoma volume, infratentorial localization, deep supratentorial hemorrhage, and lower Glasgow coma scale (GCS) scores [3, 4]. Treating patients with ICH is clinically challenging, and there is a shortage of proven treatments that improve functional outcomes [5].

Do-not-attempt-resuscitation (DNAR) decisions are predetermined to abstain from cardiopulmonary resuscitation in case of cardiac arrest [6]. Early DNAR decisions, commonly defined as issued within 48 h from admission, for ICH patients are associated with an increased mortality risk [7, 8] and a risk of less active treatment compared with patients without an early DNAR decision [9]. The consequences of early DNAR decisions on mortality led to changes in the American Guidelines for the Management of Spontaneous ICH in 2007 [10]. The current recommendation is to avoid DNAR decisions before the second day of full hospitalization [11]. However, there are no specific recommendations regarding DNAR decisions in patients with ICH in Swedish guidelines. DNAR decisions have been reported to be associated with higher age [9, 12], female sex [12], comorbidity [12], high income [12], intraventricular hemorrhage [8, 9], midline shift, and a higher National Institutes of Health Stroke Scale (NIHSS) score on arrival [9].

DNAR-decisions in patients with infratentorial or large supratentorial ICH pose a clinical and ethical challenge due to the severe prognosis. Previous studies of DNAR decisions following ICH did, however, not focus on patients with the most severe prognosis [8, 9, 13]. In this study, we examine the frequency and associated factors of DNAR decisions in a population with infratentorial or large supratentorial ICH (≥30 mL). In addition, we aim to investigate differences in complications and treatments between patients with and without a DNAR decision, and whether the DNAR decisions were associated with one-year all-cause mortality rates. To the best of our knowledge, the consequences of DNAR decisions in this subgroup have not been previously investigated.

## Methods

### Patient selection

This longitudinal, register-based, observational study included all patients with infratentorial or large supratentorial (≥30 mL) ICH, treated at three comprehensive stroke units, within the Sahlgrenska University Hospital in Gothenburg, Sweden, between 1 November 2014 and 30 June 2019. Sahlgrenska University Hospital has a catchment area of 800,000 inhabitants and is the only hospital in the region with a neurosurgical department. The neurosurgical department has a catchment area of 1.8 million inhabitants.

All patients were identified in the local Väststroke Quality Register, held by Sahlgrenska University Hospital. Additional data were collected retrospectively from medical records, the National Stroke Register in Sweden, the Longitudinal Integration Database for Health Insurance and Labor Market Studies, the National Patient Registry (NPR), and the Swedish Cause of Death Register. The data were merged by the National Board of Health and Welfare using Swedish personal identification numbers. Exclusion criteria comprised patients with restricted medical records and those whose care was initiated outside the region. Follow-up of all-cause mortality continued for 1 year after the incident ICH for all patients.

### DNAR decisions

DNAR decisions regard initiation of cardiopulmonary resuscitation in case of cardiac arrest, and does not indicate withdrawal of other care or treatment limitations in Sweden [6]. A DNAR decision must be taken by a licensed medical doctor well acquainted with the patient’s status and medical history, in consultation with another licensed healthcare professional. If possible, it must be discussed with both the patient and his or her next-of-kin. The DNAR decision must be clearly documented in the medical records [14]. If no DNAR decision is mentioned in the medical records, the patient is to receive cardiopulmonary resuscitation in case of cardiac arrest.

### Data collection

Data collection from electronic medical records was conducted using a standardized procedure. We first searched specific notes or modules for the relevant information. Second, a search was performed using the search function and pre-specified search terms. Third, if the relevant information was not clearly stated, an interpretation of the written notes was relied on, when possible.

Information concerning DNAR decisions, palliative care, living arrangements, friends or family present at the hospital, complications, and treatments were collected within 14 days after hospital arrival. Physiotherapist and occupational therapist evaluations were recorded, if performed within the first 14 days. Physiotherapist and occupational therapist evaluations should be performed on all stroke patients without referral within 48 h according to Swedish guidelines. Assessment of consciousness scores, typically documented using the reaction level scale, were collected from the medical records during the first 7 days. These scores were converted into GCS scores [15].

Being dependent was defined as receiving home care services, nursing, or living at a nursing home prior to stroke. Physical activity was defined as regular light physical activity for at least 4 h weekly [16]. Income was defined as the total income the year prior to stroke, and was divided into tertiles. Educational levels were trichotomized as follows: < 10 years (primary school), 10–12 years (secondary school), and > 12 years (postsecondary or university education). Comorbidity data were collected from the NPR and used to calculate Charlson comorbidity index (CCI) scores [17]. The CCI scores were categorized as follows: no comorbidity (0 points), mild comorbidity (1-2 points), and severe comorbidity (> 2 points).

Hematoma volume and location data were collected from initial computed tomography or magnetic resonance imaging scans on hospital admission. To calculate intraparenchymal hematoma volume, the ABC/2 formula was used. The ABC/2 formula multiplies the longest hemorrhage diameter (A) with the perpendicular diameter (B) and the number of slices multiplied by the slice thickness (C). Ventricular breakthrough was registered, but not included in the calculation of hematoma volume. Midline shift was determined as deviance > 3 mm.

### Statistical analysis

Multiple imputation by chained equations (MICE) was used to handle missing observations in the dataset. The following variables were imputed: being dependent (5.1% missing), living situation (3.2% missing), presence of next-of-kin at hospital (0.6% missing), pre-stroke physical activity (14.6% missing), and education (1.0% missing). The variables were imputed separately for patients with and without DNAR decisions. Descriptive data are presented as median with interquartile range (IQR) or mean with standard deviation (SD) for continuous variables, and number and percentage for categorical variables. Binary logistic univariable and multivariable regression models were conducted to predict DNAR decisions. As next-of-kin were present at the hospital for almost all patients (96.4%), and only one patient with no DNAR received palliative care (0.6%), these variables were excluded from the analyses. Multicollinearity was assessed using Spearman’s rank correlation for ordinal variables, and Phi coefficient for nominal variables. Goodness of fit was determined using a Hosmer and Lemeshow test (*p* > 0.05 indicated good fit). The explained variance of the models was determined using Nagelkerke’s *R*^2^. Area under the curve was assessed using receiver operating characteristic curves. Kaplan-Meier curves were used to visualize cumulative survival rates. A multivariable Cox proportional hazards regression was used to model the risk of one-year mortality. Schoenfeld residuals were used to test independence between residuals and time. All statistical tests were two-tailed and interpreted at a significance level of 0.05.

## Results

**Fig. 1:**
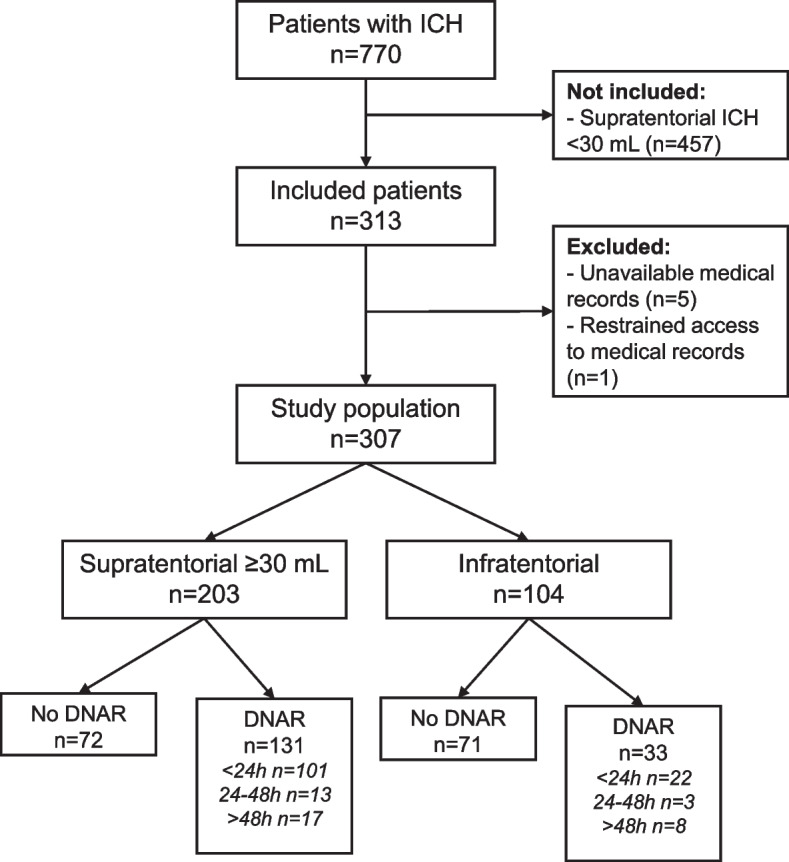
Flowchart of inclusion procedure. Abbreviations: DNAR, do-not-attempt-resuscitation; ICH, intracerebral hemorrhage

During the study period, 770 patients were treated for ICH. After reviewing the medical records, 307 patients were included (Fig. 1). There were 208 (68%) patients with an ICH volume ≥ 30 mL, and 104 (34%) patients with an infratentorial ICH. Median volumes are shown in Fig. 1. Baseline characteristics are presented in Table 1. In total, 164 (53%) patients received a DNAR decision, most of which (75%) were made during the first 24 h. Almost all patients were treated at a stroke unit (71%) or intensive care unit (38%). Only one patient was treated at a general ward.

**Table 1:** Study population characteristics according to DNAR decisions

	No DNAR decision ***n*** = 143	DNAR decision ***n*** = 164
Baseline characteristics		
Female sex, n (%)	82 (57.3)	66 (40.2)
Age, median (IQR)	68 (57-77)	83 (76-87)
Physical activity, n (%)	64 (44.8)	29 (17.7)
Born in Sweden, n (%)	109 (76.2)	131 (79.9)
Living alone, n (%)	53 (37.1)	90 (54.9)
Dependent, n (%)	18 (12.6)	70 (42.7)
Next-of-kin at hospital, n (%)	138 (96.5)	158 (96.3)
Palliative care, n (%)	1 (0.6)	97 (59.1)
Education, n (%)		
< 10 years	39 (27.3)	69 (42.1)
10-12 years	74 (51.7)	66 (40.2)
> 12 years	30 (21.0)	29 (17.7)
Income, n (%)		
Low	46 (32.2)	60 (36.6)
Medium	40 (28.0)	67 (40.9)
High	57 (39.9)	37 (22.6)
Charlson comorbidity index, n (%)		
0 points	84 (58.7)	75 (45.7)
1-2 points	32 (22.4)	55 (33.5)
> 2 points	27 (18.9)	34 (20.7)
NIHSS, median (IQR)	5 (1-16)	21 (15-25)
Midline shift, n (%)	72 (50.3)	121 (73.8)
Intraventricular hemorrhage, n (%)	51 (35.7)	108 (65.9)
Complications within 14 days from ICH onset		
New stroke, n (%)	17 (11.9)	24 (14.6)
Acute coronary syndrome, n (%)	1 (0.7)	3 (1.8)
Pulmonary embolism, n (%)	1 (0.7)	0 (0)
Deep vein thrombosis, n (%)	1 (0.7)	0 (0)
Pneumonia, n (%)	24 (16.8)	29 (17.7)
UTI/pyelonephritis, n (%)	14 (9.8)	8 (4.9)
Infection, n (%)	27 (18.9)	22 (13.4)
Sepsis, n (%)	5 (3.5)	4 (2.4)
Pressure ulcers, n (%)	4 (2.8)	8 (4.9)
Anxiety, n (%)	36 (25.2)	47 (28.7)
Pulmonary edema, n (%)	4 (2.8)	8 (4.9)
Gastrointestinal ulcers, n (%)	9 (6.2)	19 (11.6)
Bone fracture, n (%)	3 (2.1)	1 (0.6)
Treatment within 14 days from ICH onset		
Thrombosis prophylaxis, n (%)	82 (57.3)	52 (31.7)
Oral antibiotics, n (%)	16 (11.2)	7 (4.3)
Intravenous antibiotics, n (%)	49 (34.3)	51 (31.1)
Oral paracetamol, n (%)	113 (79.0)	116 (70.7)
Intravenous paracetamol, n (%)	68 (48.3)	61 (37.2)
Physiotherapist evaluation, n (%)	141 (98.6)	73 (44.5)
Occupational therapist evaluation, n (%)	126 (88.1)	64 (39)

*Abbreviations*: *DNAR* Do-not-attempt-resuscitation, *IQR* Interquartile range, *NIHSS* National Institute of Health stroke scale, *ICH* Intracerebral hemorrhage, *UTI* Urinary tract infection

Frequencies of in-hospital complications were similar between patients with and without a DNAR decision (Table 1). Patients without DNAR decisions received thrombosis prophylaxis and oral antibiotics more frequently; they were also evaluated by a physiotherapist or occupational therapist twice as often (Table 1). Factors associated with DNAR decisions from the univariable, and multivariable binary regression models are reported in Table 2. Only age, pre-stroke dependency, NIHSS, and intraventricular hemorrhage remained significantly associated in adjusted models.

**Table 2:** Binary univariable and multivariable logistic regression analyses for the prediction of DNAR decisions

	Univariable analyses		Multivariable analysis	
	OR (95% CI)	***p***-value	aOR (95% CI)	***p***-value
Female sex	2.00 (1.27–3.15)	0.003	0.88 (0.40–1.95)	0.747
Age	1.10 (1.07–1.12)	< 0.001	1.14 (1.09–1.18)	< 0.001
Physical activity	0.27 (0.16–0.45)	< 0.001	0.85 (0.37–1.96)	0.702
Born in Sweden	0.81 (0.47–1.39)	0.440	0.53 (0.20–1.42)	0.204
Living alone	2.07 (1.31–3.27)	0.002	1.05 (0.42–2.59)	0.921
Elderly care services	5.17 (2.89–9.26)	< 0.001	4.06 (1.41–11.70)	0.009
Education < 10 years	Reference		Reference	
Education 10–12 years	0.50 (0.30–0.84)	0.009	1.13 (0.48–2.68)	0.775
Education > 12 years	0.55 (0.29–1.04)	0.066	0.85 (0.27–2.66)	0.774
Low income	Reference		Reference	
Medium income	2.01 (1.14–3.53)	0.015	1.24 (0.48–3.24)	0.658
High income	2.58 (1.46–4.56)	0.001	0.501 (0.16–1.56)	0.232
Charlson comorbidity index 0	Reference		Reference	
Charlson comorbidity index 1–2	1.93 (1.13–3.29)	0.017	1.30 (0.52–3.27)	0.571
Charlson comorbidity index > 2	1.41 (0.78–2.55)	0.256	0.91 (0.32–2.58)	0.856
NIHSS	1.16 (1.12–1.20)	< 0.001	1.27 (1.19–1.35)	< 0.001
Midline shift	2.78 (1.72–4.48)	< 0.001	0.80 (0.35–1.83)	0.596
Intraventricular hemorrhage	3.48 (2.17–5.57)	< 0.001	2.53 (1.14–5.65)	0.023

Hosmer and Lemeshow test: χ^2^ = 9.27, *p* = 0.32; Nagelkerke *R*^2^ 0.72

*Abbreviations*: *aOR* Adjusted odds ratio, *CI* Confidence interval, *DNAR* Do-not-attempt-resuscitation, *NIHSS* National Institute of Health stroke scale, *OR* Odds ratio

**Fig. 2:**
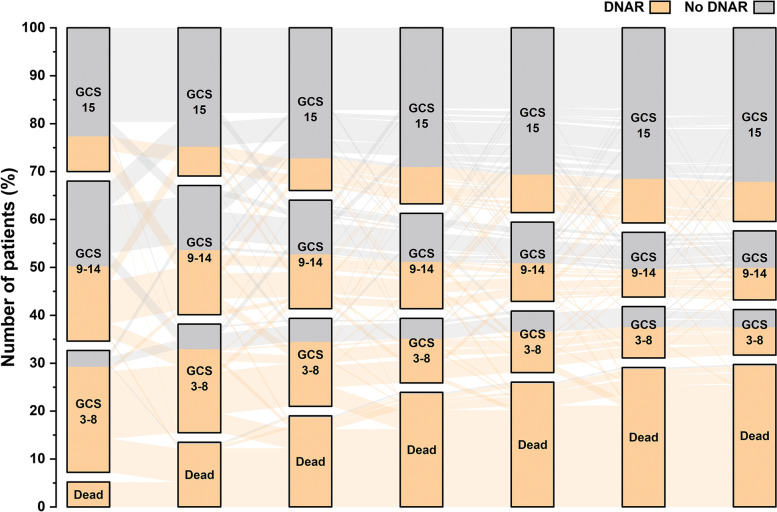
Intraindividual day-to-day change in GCS during the first week after ICH onset. Abbreviations: DNAR, do-not-attempt-resuscitation; GCS, Glasgow coma scale; ICH, intracerebral hemorrhage

Intra-individual day-to-day changes in GCS scores during the first week after admission are presented in Fig. 2. All but one patient who died the first week had a DNAR decision, whereas most patients with a GCS score of 15 had no DNAR decision. As the first week progressed, the number of patients with a GCS score of 15 increased. Most patients with GCS scores of 3–8 had a DNAR- decision.

**Fig. 3:**
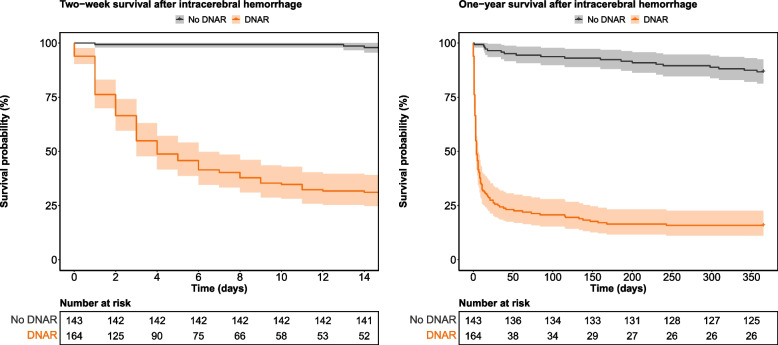
Cumulative survival curves with 95% confidence intervals according to DNAR decisions at 2 weeks, and 1 year after ICH-onset. Abbreviations: DNAR, do-not-attempt-resuscitation; ICH, intracerebral hemorrhage

Two weeks post-admission, only 52 (32%) patients with DNAR decisions were alive with a cumulative survival probability of 0.31 (95% confidence interval [CI] 0.25–0.39) compared to 141 (99%) patients without DNAR decisions with a cumulative survival probability of 0.98 (95% CI 0.96–1.00). One year after incident ICH, the cumulative survival probability was 0.16 (95% CI 0.11–0.23) for patients with DNAR decisions, and 0.87 (95% CI 0.81–0.92) for patients without DNAR decisions (Fig. 3). The one-year all-cause risk of mortality was associated with a DNAR decision, higher age, comorbidities, a greater NIHSS score and increasing hematoma volume. Living alone and thrombosis prophylaxis were associated with a decreased risk of mortality (Table 3).

**Table 3:** Multivariable Cox proportional hazards model for associations between covariates and One-year-mortality

	One-year mortality		
	N (%)	aHR (95% CI)	***p***-value
DNAR decision	164 (53)	5.68 (3.20–10.08)	< 0.001
Female sex	148 (48)	1.15 (0.79–1.67)	0.471
Age, mean (SD)	74 (15)	1.03 (1.01–1.05)	0.001
Physical activity	93 (30)	1.34 (0.84–2.15)	0.222
Born in Sweden	240 (78)	0.81 (0.53–1.24)	0.325
Living alone	143 (47)	0.52 (0.33–0.82)	0.005
Dependent	88 (29)	1.30 (0.83–2.04)	0.255
Education < 10 years	108 (35)	Reference	
Education 10–12 years	140 (46)	1.07 (0.71–1.59)	0.754
Education > 12 years	59 (19)	1.14 (0.68–1.91)	0.614
Low income	106 (34)	Reference	
Medium income	107 (35)	1.28 (0.82–1.99)	0.282
High income	94 (31)	0.90 (0.54–1.53)	0.721
Charlson comorbidity index 0	159 (52)	Reference	
Charlson comorbidity index 1–2	87 (28)	1.68 (1.14–2.48)	0.009
Charlson comorbidity index > 2	61 (20)	2.13 (1.36–3.33)	< 0.001
NIHSS, mean (SD)	14 (9)	1.11 (1.08–1.15)	< 0.001
Midline shift	193 (63)	0.82 (0.51–1.33)	0.429
Deep location	78 (25)	Reference	
Lobar location	125 (41)	0.78 (0.51–1.21)	0.267
Infratentorial location	104 (34)	1.09 (0.63–1.91)	0.753
Hematoma volume, mean (SD)	56 (52)	1.01 (1.00–1.01)	< 0.001
Intraventricular haemorrhage	159 (52)	1.01 (0.69–1.49)	0.953
Thrombosis prophylaxis	134 (44)	0.35 (0.23–0.51)	< 0.001

Multivariable Cox proportional hazards model including all covariates in the table. Global correlation between the Schoenfeld residuals and survival time: χ^2^ = 23.3, *p* = 0.273; Log rank test 354.4; *p* < 0.001

*Abbreviations*: *aHR* Adjusted hazards ratio, *CI* Confidence interval, *DNAR* Do-not-attempt-resuscitation, *NIHSS* National Institute of Health stroke scale, *SD* Standard deviation

## Discussion

In this longitudinal, register-based, observational study, we investigated the issuing of DNAR decisions in patients with infratentorial or large supratentorial ICHs. Additionally, we explored predictors of DNAR decisions and consequences in terms of in-hospital complications, treatments, and one-year mortality. Patients with infratentorial or large supratentorial ICHs have a particularly severe prognosis with high mortality, and very few fully recover [3, 18]. Previous studies on DNAR decisions following ICH and their impact of survival over time are scarce, and the consequences of DNAR decisions in this subgroup have not been investigated.

We found that half of all patients with infratentorial or large supratentorial ICHs received a DNAR decision. Most (75%) decisions were issued within the first 24 h. The proportion of DNAR decisions in our study was higher compared with those in previous studies, where 35–41% of patients received a DNAR decision following ICH [8, 9]. However, the frequency of DNAR decisions within 24 h was similar, 73% in a Finnish study [9], and 88% in a study from Sweden [8]. In contrast, in a recent American study, only 20% of patients received an early DNAR decision [19]. The higher frequency in our study may be explained by a study population with more severe stroke cases due to the exclusion of patients with small supratentorial ICHs, and the inclusion of patients from hospitals connected to a regional neurosurgical department.

One-year mortality was higher among patients with DNAR decisions, which accords with previous research [9]. The greatest difference between patients with and without DNAR decisions was seen within the first 2 weeks when most of the patients with DNAR decisions died. The majority of patients who died had low GCS scores at admission. Impaired level of consciousness has been associated with higher mortality, mediated through withdrawal of life sustaining treatment [20]. However, not all patients with DNAR decisions in our study received palliative care. It is notable that all but one of the study patients who died within 1 week had a DNAR decision, despite a similar frequency of complications compared to patients without DNAR decisions. These findings may represent that clinicians intuitively identify patients with the highest risk of dying early after ICH.

Higher age, pre-stroke dependency, higher NIHSS scores, and intraventricular hemorrhage were independent factors associated with DNAR decisions in this study. Premorbid functional status has previously been reported as an independent predictor for DNAR decisions following ICH [21]. In our study, being dependent involved a variety of care levels such as living at home with help from homecare services or home health aides to living at a nursing home. Moving to an institution for older adults might be seen as a surrogate marker for imminent death, and up to one-third of older adults may die within 6 months from moving into a nursing home [22]. Living alone was associated with decreased mortality in the current study, which is in contrast to prior research showing that people living alone may be at higher risk of post-stroke mortality [23, 24]. In comparison, the median age in this study was considerably higher, and living alone may therefore be a surrogate marker for good health rather than lacking of social support.

In line with our study results, previous research have found associations between DNAR decisions and higher age, greater NIHSS score, and intraventricular hemorrhage [8, 9, 12]. These are also previously known factors associated with post-stroke mortality [3, 25]. Moreover, we observed that higher age, greater NIHSS scores, larger hematoma volumes, comorbidities and DNAR decisions, were associated with increased mortality in infratentorial and large supratentorial ICHs. Other studies have also reported associations between DNAR decisions and ICH mortality [7–9]. Further, significant comorbidities have also been associated with mortality following ICH [7].

Overall, patients with and without DNAR decisions received similar treatments, although thrombosis prophylaxis was administered more often to patients without DNAR decisions, similar with previous research [9]. The use of thrombosis prophylaxis in ICH patients is controversial; the risk of re-bleeding must be weighed against the risk of embolism. A review of randomized control trials for thrombosis prophylaxis showed inconclusive results [26], whereas a more recent retrospective cohort analysis showed no re-bleeding with early thrombosis prophylaxis [27]. Given these varying findings, the use of thrombosis prophylaxis for patients with ICH requires further investigation. Ultimately, mortality rates were found to be high in this study population, and DNAR decisions appeared to mediate this association. DNAR decisions, however, remains a clinical challenge in patients with severe ICH. Practicing physicians as well as future guidelines should consider DNAR decisions carefully in relation to the subgroup of patients with the most severe ICH.

## Strengths and limitations

In this large sample, we investigated the issuing of DNAR decisions, as well as long- and short-term consequences of such decisions in patients with infratentorial or large supratentorial ICHs. A strength of our study design was that nor physicians or patients were aware of our research, and the DNAR decision-making process was not influenced by the study. We were able to investigate multiple factors concurrently; however, we could not control the information recorded in medical records and could not monitor missing data. Moreover, the data were interpreted by two observers, namely, the clinician recording the medical records initially and by the researchers during the data collection. To minimize the risk of bias when classifying the data, we established a pre-specified protocol prior to record reviewing. Another study strength was the merging of data with national Swedish registries, which are validated and have high coverage. Through combining the data collected from medical records with several registers, we were able to reduce the amount of missing data and include several predictive variables. Lastly, the tax-funded health care in Sweden, equally accessible to everyone decrease the risk of selection bias.

One study limitation was that we included patients from three centers within one university hospital, in addition to patients who had been transferred from surrounding hospitals in need of neurosurgical care. Therefore, the study sample may not be representative of the general population with severe ICH. In particular, our findings may not be representative for ICH populations in areas with limited health care resources, or with another social, cultural or ethical stand on DNAR decisions. Another limitation is the possibility of unobserved confounders. Many factors can influence DNAR decisions and survival, which make it challenging to account for all potentially confounding factors. Furthermore, we were not able to collect information concerning the motivations for making DNAR decisions due to inconsistency in documentation within the medical records. Finally, our retrospective, observational research study design entailed limited internal validity due to potential biases in terms of patient selection, missing data, measurement of outcomes, and in classifying interventions.

## Conclusions

Half of all study patients with infratentorial or large supratentorial ICHs received a DNAR decision, most within 24 h of admission. Significant predictors for DNAR decisions were higher age, pre-stroke dependency, stroke severity, and intraventricular hemorrhage. Patients with and without DNAR decisions did not differ in terms of in-hospital complications; however, patients without DNAR decisions more frequently received thrombosis prophylaxis, oral antibiotics, and rehabilitative evaluations. The one-year mortality rate was considerably higher in patients with a DNAR decision, although most of the deaths in this group occurred within the first 2 weeks. The risk of one-year mortality was associated with higher age, comorbidities, greater stroke severity, increased hematoma volume, and DNAR decisions. Thrombosis prophylaxis and living alone were associated with a lower risk for mortality.

## Data Availability

Data that support the findings of this study are available on request from the Professor Katharina Stibrant Sunnerhagen (ks.sunnerhagen@neuro.gu.se). The data are not publicly available due to privacy and ethical restrictions.
